# Place Is Power: Investing in Communities as a Systemic Leverage Point to Reduce Breast Cancer Disparities by Race

**DOI:** 10.3390/ijerph19020632

**Published:** 2022-01-06

**Authors:** Matthew Jay Lyons, Senaida Fernandez Poole, Ross C. Brownson, Rodney Lyn

**Affiliations:** 1 WellStar College of Health and Human Services, Kennesaw State University, Kennesaw, GA 30144, USA; mlyons30@kennesaw.edu; 2 Office of the President, California Breast Cancer Research Program, University of California, Oakland, CA 94607, USA; senaida.poole@ucop.edu; 3 Prevention Research Center in St. Louis, Brown School, Washington University in St. Louis, St. Louis, MO 63130, USA; rbrownson@wustl.edu; 4 Division of Public Health Sciences, Department of Surgery, Washington University School of Medicine, Washington University, St. Louis, MO 63110, USA; 5 Alvin J. Siteman Cancer Center, Washington University School of Medicine, St. Louis, MO 63110, USA; 6 School of Public Health, Georgia State University, Atlanta, GA 30302, USA

**Keywords:** review paper, breast cancer, racism, health disparities, residential segregation, systems theory, community engagement

## Abstract

Racial disparities in breast cancer present a vexing and complex challenge for public health. A diverse array of factors contributes to disparities in breast cancer incidence and outcomes, and, thus far, efforts to improve racial equity have yielded mixed results. Systems theory offers a model that is well-suited to addressing complex issues. In particular, the concept of a systemic leverage point offers a clue that may assist researchers, policymakers, and interventionists in formulating innovative and comprehensive approaches to eliminating racial disparities in breast cancer. Naming systemic racism as a fundamental cause of disparities, we use systems theory to identify residential segregation as a key leverage point and a driver of racial inequities across the social, economic, and environmental determinants of health. We call on researchers, policymakers, and interventionists to use a systems-informed, community-based participatory approach, aimed at harnessing the power of place, to engage directly with community stakeholders in coordinating efforts to prevent breast cancer, and work toward eliminating disparities in communities of color.

## 1. Introduction

There are significant racial disparities in breast cancer incidence, care, and outcomes, with Black women experiencing higher incidence of aggressive breast cancer subtypes, lower quality of care, and higher mortality rates than white women [1,2,3]. These disparities persist despite efforts to eliminate them. An increasing number of scholars and scientists identify systemic racism as the fundamental cause of health disparities [4,5,6]. This causal effect is exerted through numerous interrelated pathways, including residential segregation, economic deprivation, healthcare quality and access, social and environmental exposures, and environmentally conditioned health behavior (e.g., smoking). In order to be effective, efforts to prevent breast cancer and eliminate disparities must therefore acknowledge and work to address racism *as a system*. This approach differs from traditional intervention paradigms, which seek to isolate factors in a linear causal chain resulting in a single health outcome, in that it works from the assumptions that multiple interrelated causes can exert a multiplicative influence over and above the sum of their effects, that numerous outcomes are likely to trace back to the same interrelated set of causes, and that disruptions in one subsystem will likely not change outcomes. Focus is therefore shifted from finding a single cause to finding those subsystems, or nodes in the network of related causes, that mediate the relationships between numerous other subsystems and the outcome(s) of interest. Those component subsystems that are necessary for the maintenance of systemic equilibrium can be understood as leverage points [4].

With respect to health disparities generally, and breast cancer disparities specifically, residential racial segregation is one such leverage point. It has historically produced inequities in access to opportunities and resources [7]. Place of residence largely determines access to high quality education, which influences educational attainment and subsequent labor market opportunities [8]. Additionally, place of residence affects access to credit markets, housing, health care, and the quality and nature of interactions with law enforcement and the criminal justice system [4]. People of color in the United States continue to be concentrated to a great degree in communities with lower levels of resources and opportunities relative to white communities, with resulting inequities and disparities in outcomes associated with health and well-being. Residential racial segregation has facilitated inequitable access and exposure with regard to the social, economic, and environmental determinants of health. It has served as a fulcrum—a leverage point— in the race discrimination system, influencing the operation of every major subsystem, and producing racial inequities and disparities. In this article, we will outline an approach to harnessing the power of place, such that it may serve as a leverage point to amend, rather than contribute to racial disparities in breast cancer.

We begin with an overview of Black/white racial breast cancer disparities, followed by a brief outline of the state of the scientific literature on individual risk factors for, and protective factors against, breast cancer, with particular focus on those risk and protective factors that disproportionately impact historically marginalized groups. We then discuss various potential mechanisms by which racial discrimination may exert a causal influence on breast cancer disparities by conditioning the population distribution of these risk and protective factors, with the caveat that these mechanisms are not yet well understood. In order to re-conceptualize this web of interrelated influences in a more actionable framework, we echo other scholars and scientists in identifying systemic racism as the underlying cause that links all of the interrelated mechanisms of disparity together. We use concepts from systems theory to demonstrate that residential segregation is a key leverage point in the racial discrimination system. We then put forth a systems-oriented, community-engaged approach to breast cancer prevention and disparities research and outline opportunities for researchers, policymakers, and interventionists to harness the power of place by partnering directly with community stakeholders in coordinating breast cancer prevention efforts in under-resourced minority communities.

While a detailed description of Black/white racial breast cancer disparities is beyond the scope of this paper, there is a large body of scientific data indicating that breast cancer and its consequences are differentially distributed by race. Because these differences adversely impact marginalized communities, and are related to injustice, they are called health disparities. In our usage, therefore, the term disparity should be taken to mean more than simply difference, but a difference which has its source in inequity or injustice. Racial breast cancer disparities can be understood as falling into three broad categories: disparities in incidence, disparities in care, and disparities in outcome. Historically, Black women have had lower rates of breast cancer than white women. However, recent literature indicates that breast cancer incidence is rising among Black women, and in 2012, overall breast cancer incidence for Black women reached levels equaling those of White women [2]. Though this on its own does not illuminate an absolute Black/white breast cancer disparity, it does show a disparity in change over time wherein Black women do not share equally in improvements occurring across the broader population. Further, in a study of 375,761 cases of breast cancer, Black women were more likely than white women to be diagnosed with triple-negative breast cancer, which is more aggressive and difficult to treat than more common breast cancer subtypes [9]. In that same study, the overall incidence rate for breast cancer among women less than 44 years old was highest among Black women.

Compared with white women, Black women disproportionately experience longer waiting periods after abnormal screening, treatment delays, lower likelihood of receiving guideline-concordant cancer care [1], lower quality of care [10], and early treatment termination [11], which has been linked to negative outcomes. Though mortality rates for breast cancer have steadily decreased in recent decades, outcomes have improved less for Black women than for white women [12]. For sex-specific cancers, including breast cancer, racial disparities in survival have been shown in a randomized trial of nearly 20,000 subjects to remain significant when controlling for other demographic factors, as well as clinical and treatment variables [13]. In fact, as breast cancer incidence among Black women has increased, mortality rates for breast cancer have also continued to be higher among Black women than among any other racial group [3,14]. In 2012, breast cancer mortality among black women was 42% higher than it was among white women [2].

## 2. The Fundamental Causes of Breast Cancer Disparities

Breast cancer disparities persist despite the development and implementation of numerous interventions seeking to eliminate them [15]. There are several comprehensive and wide-ranging reviews of the current state of scientific research on factors that contribute to breast cancer incidence, outcomes, and disparities [3,16,17]. Overall, this literature indicates that risk and protective factors are distributed differently by race, with racial minorities more likely than whites to be exposed to many risk factors. Specifically, it points to the need for prevention efforts aimed at *reducing* alcohol consumption, tobacco use, and chronic stress, as well as chemical, occupational, and ionizing radiation exposures, and *promoting* breastfeeding, healthy diet patterns, physical activity, and healthy vitamin D levels [3,16,17]. Though our ultimate aim is to highlight the complex interrelationships between the environmental determinants of breast cancer outcomes, we will begin with a brief discussion of some individual risk and protective factors that are differentially distributed by race. We will then turn our attention to the ways in which racial residential segregation ensures the inequitable distribution of these determinants at the population level.

### 2.1. Risk and Protective Factors for Breast Cancer

Alcohol is a known risk factor for breast cancer, and there is a dose response relationship between the two, with higher consumption corresponding to greater breast cancer risk [18]. Though there are some conflicting results, there is limited evidence that high levels of alcohol consumption are associated with greater breast cancer risk among Black women than for any other racial group, and that higher levels of alcohol consumption increase risk of more aggressive subtypes of breast cancer, which also disproportionately impact Black women [19,20].

The carcinogenic properties of tobacco smoke are well known. Though smoking is more strongly associated with cancers of the mouth, throat, and lungs, it is also a risk factor for breast cancer, particularly among women who started smoking at a young age or before carrying a pregnancy to term [16,17]. Smoking is a risk factor for luminal breast cancer in particular, and there is some evidence that that risk is especially elevated for Black women [21]. Further, another study found that though race and class were not a predictor of tobacco spending, vulnerable populations may experience worse breast cancer outcomes attributed to tobacco addiction than others [22].

Environmental pollutants and toxic exposures account for between 7% and 19% of the world’s cancer cases [16,23]. Exposures to toxic substances in the air, in the water, and through industrial agricultural pesticides contribute significantly to those figures, and there is burgeoning evidence that people of color are disproportionately impacted by those exposures [24]. There is a broad array of chemical compounds in common household products that are of concern for breast cancer preventionists, including: bisphenol A (BPA); heavy metals, such as cadmium; polybrominated diphenyl ethers (PBDEs, AKA flame retardants); phthalates; alkylphenols; per- and polyfluoroalkyls (PFAs); pesticides; herbicides; solvents; aromatic amines; and parabens [16]. There are various mechanisms by which these compounds may increase breast cancer risk, including endocrine disruption, promotion of tumor growth, and adverse epigenetic impacts. There is an emerging body of evidence to suggest that people of color are exposed to carcinogens in consumer products more frequently and in higher doses than whites [25,26].

Ionizing radiation is a risk factor for various cancers, including breast cancer, and a common mechanism of exposure is in medical imaging, such as CT scans and X-Rays [16]. One area of potential concern regarding racial disparities in breast cancer incidence is that, though Black women are less likely to receive testing for BRCA1/2 mutations, exposure to ionizing radiation through medical diagnostics was found in at least one study to increase risk of breast cancer by 90% among women with that mutation [27].

Occupational factors may relate with breast cancer incidence and outcomes through numerous possible mechanisms, including: “chemical exposure; stress, including around job security and fair wages, threats or acts of sexual and physical violence, and lack of power to advocate for oneself; challenges with time and accommodation for breastfeeding; light-at-night exposure; and many other issues” [16] (p. 204). These factors are of particular concern for women of color, who likely experience added stressors related to discrimination which may exacerbate their deleterious effects [28,29].

Though the biological mechanisms linking breast cancer and diet are not well understood, the extant literature indicates that diet can function both as a risk factor and as a protective factor for breast cancer. Though some conflicting findings complicate the picture, the bulk of relevant studies show a positive association between consumption of red and processed meat and breast cancer [30,31,32]. A recent meta-analysis also found a positive association between adolescent fat intake and breast cancer risk later in life [33].

On the other hand, the preponderance of scientific evidence suggests that healthy eating habits, such as the Mediterranean diet, have a protective effect against breast cancer, and improve overall health [16]. Due to the relatively lower quality of food sources in low-income communities of color, these diet-related risk factors may contribute significantly to disparities [34].

A recent meta-analysis including 13,907 breast cancer cases from 27 studies on the relationship between breastfeeding and breast cancer found that there was an inverse association between the two, with longer breastfeeding duration related with lower relative risk compared with shorter duration (RR = 0.471, 95% CI, 0.368–0.602) [35]. At the same time, one California based study found that Black women are less likely to exclusively breastfeed their infants than white women [36]. This may be related to the fact that Black women are more likely to be employed in occupations with little to no flexibility or support services for new mothers, such as paid family leave, or experience other structural impediments related to discrimination that make breastfeeding more difficult.

Vigorous physical activity is another likely protective factor against breast cancer [15,16]. These effects have been observed in studies that focused on Black women specifically [37,38]. There are reasons to believe, however, that women of color may have lower levels of physical activity, and that this disparity in activity can be explained by the fact that they disproportionately experience structural and logistical barriers to physical activity, such as unsafe neighborhoods and a lack of access to greenspace [39,40,41].

There is some evidence to suggest that vitamin D deficiency may be related to breast cancer disparities. In one study, low levels of vitamin D were associated with an elevated risk of breast cancer of 23% in Black women [42]. Black women are at elevated risk of triple negative breast cancer, a particularly aggressive subtype, and are 10 times more likely to experience vitamin D deficiency than white women. This has led some researchers to suggest that some of that elevated risk may be attributable to vitamin D deficiency [43].

### 2.2. The Role of Social Determinants

Williams et al. note that though both racial discrimination and racial breast cancer disparities are well documented, the mechanisms that link discrimination to disparities are not well understood [3]. There are numerous plausible ways to begin tying the individual risk and protective factors together with discrimination into a coherent theoretical framework of racial breast cancer disparities. We briefly outline and assess several possibilities below. Though they are presented separately here, the causal mechanisms are not truly separable. In reality, they are deeply interconnected.

At the individual level, the positive association between low SES and disease does not generally hold for breast cancer, but striking patterns emerge when examining incidence, severity of breast cancer subtype, race/ethnicity, and SES together [3,44,45]. Discrimination can be understood as a stressor, and there is burgeoning research examining the impact of this specific type of stress as it relates to overall health. For example, experiences of discrimination have been linked to poorer health, and higher levels of biological and behavioral indicators of disease risk, such as inflammation, obesity, and smoking [46,47].

Further, there is a growing body of literature demonstrating the neurobiological impacts of stress and trauma in early life, including methylation of the nuclear receptor 3C1, “the gene that codes for the glucocorticoid receptor on the hypothalamic-pituitary-adrenal axis” [3] (p. 2141). Increased methylation in this nuclear receptor “represents a unique record of past adverse psychosocial experience,” and has been shown to be associated with numerous negative health outcomes, including breast cancer [3] (p. 2141). Further, Geronimus et al. put forth the concept of weathering to encapsulate the effect of chronic stressors experienced by Black women in America [28,29]. One example of this effect is reflected in a process of accelerated aging. By measuring telomere length, Geronimus and colleagues were able to determine that, compared with white women, Black women in their sample were biologically 7.5 years older on average. Another key concept related to stress across the life course is allostatic load, a biological measure of overall wear and tear on the body due to stress. As Williams et al. put it: “the concept of allostatic load has been used to capture the biological dysregulation across multiple physiological systems that result from the cumulative burden of repeated stressors” [3] (p. 2141). Allostatic load has been found in multiple studies to be higher in people of color than among whites, and to be associated with poor health [29].

Place of residence impacts breast cancer risk significantly. In a recent systematic review of 17 studies focusing on racial residential segregation and cancer disparities, 70% of studies showed a statistically significant association between segregation and health disparities. The authors state: “residing in segregated African-American areas was associated with higher odds of later-stage diagnosis of breast and lung cancers, higher mortality rates and lower survival rates from breast and lung cancers, and higher cumulative cancer risks associated with exposure to ambient air toxins” [48] (p. 1195). Racial residential segregation also ensures that place-based risk factors are inequitably distributed by race. Given that individual and environmental exposures to breast cancer risk factors are impacted by place of residence, it is plausible that the disparities in incidence, care, and outcomes outlined by Landrine et al. are the result of the differential distribution of these exposures between segregated neighborhoods [48]. Again, though these myriad factors are presented separately above, the upstream contributors to breast cancer disparities are not actually separable in reality.

The treatment above is not nearly complete, as it only alludes to a system of racial inequity, discrimination, and disparity that has deep historical roots and implications for nearly every domain in which health and welfare can be measured. Though there are many plausible mechanisms by which racial breast cancer disparities may arise, empirical investigation into those mechanism is in its early stages. For example, questions remain regarding the linkages between socioeconomic factors such as labor market opportunity, educational access, and credit access, indicators of health risk such as high allostatic load, and breast cancer risk. It is clear, however, that the impacts of both historical and ongoing racial discrimination are pervasive and highly interrelated. The high degree of interrelation between possible causes complicates matters for researchers seeking a parsimonious explanatory model, and interventionists seeking a small number of variables on which to focus programs. A clearer understanding of these causes and their interrelationships could aid in efforts to prevent breast cancer, and reduce disparities.

## 3. How Systems Thinking Can Identify Leverage Points

Arising out of the biological sciences, with applications across a broad range of fields, including economics, bioinformatics, meteorology, and others, systems theory is designed to model complex, interrelated networks of component subsystems. It may therefore prove useful in developing conceptual models to explain the complex and highly interrelated causes of breast cancer disparities, and to clarify why those disparities have thus far proven resistant to intervention. Most discussions of breast cancer disparities and other health inequities are confined to the healthcare or public health systems, and stop short of articulating the broader, more fundamental problem of systemic racism that contributes to inequities across a diverse array of outcomes. In the following section, we will outline a framework for research and intervention in breast cancer disparities that is explicitly informed by an acknowledgement of the interlinked nature of disparities across multiple domains, including large-scale policy frameworks, local-level social and environmental factors, and the biological sequelae of discrimination [4].

Much like racial breast cancer disparities themselves, the biological and social contributors underlying those disparities do not arise in a vacuum, but out of a long history of systemic racism and discrimination. There are significant disparities between Black and white Americans in the criminal justice system, including policing practices, incarceration, and jury participation, which have been linked not only to explicit legal discrimination, but also to implicit bias [49]. Residential segregation, which persists today despite no longer having the force of law, is a significant driver of negative outcomes across numerous domains, including health, through its impact on educational access, employment opportunities, and exposure to environmental hazards [7].

Systemic racism, and racial residential segregation in particular, also ensure the inequitable distribution of each of the risk and protective factors for breast cancer outlined above. For example, place of residence impacts the quality of available foods, exposure to environmental toxins through residential and occupational sources, likelihood of tobacco and alcohol use, and opportunities for physical activity (through access to safe outdoor spaces) [15,22,35,40,41,42,50,51,52]. Further, though socioeconomic status, education, and employment status are each associated with both race and health outcomes, racial disparities in health outcomes persist even among Black Americans who are high SES, highly educated, and gainfully employed [53]. This suggests that racism contributes independently to health disparities over and above the effects of these mediators.

There is increasing scholarly and scientific consensus that racism itself is a fundamental cause of health disparities. Yearby argues that the social determinants of health framework articulated in Healthy People 2020 is insufficient precisely because it does not give a primary place to racism as an upstream factor contributing to all other social determinants [6]. Williams et al. provide an overview of the social context of breast cancer disparities among Black women, outlining the burgeoning body of research on the biological effects of discrimination which ultimately lead to elevated breast cancer risk [3]. Reskin gives a broad outline of racial discrimination in American society, and makes a compelling case that each of the subsystems in which disparities exist are connected by the broader system of racism within which they are situated [4]. According to her model, racial disparities in outcomes across numerous domains are the result of a single, integrated system of racial discrimination. She argues that ad hoc, sector-specific interventions to reduce disparities will likely fail to disrupt the systemic equilibrium that maintains inequity due to the systemic property of robustness. This literature indicates that, in order for racial breast cancer disparities to be eliminated, researchers, policymakers, and interventionists must address racism *as a system*. In the following section, we will introduce key concepts from systems theory, and discuss ways in which they can be used as tools to reconceptualize racial breast cancer disparities in light of the system of racial discrimination within which they arise.

### 3.1. Emergence

Established systems exhibit the property of emergence. In a network of interrelated subsystems, the system as a whole will begin to exert an influence over and above the sum of the additive effects of those component subsystems. As Reskin explains, within the race discrimination system, this means that, in addition to the effects of individual experiences of discrimination and explicitly traceable instances of institutional racial bias, the system of racial discrimination itself exerts influence in maintaining the equilibrium of racial disparity [4]. In other words, the interrelationships, or feedback loops, between factors like Adverse Childhood Experiences (ACEs) and childhood poverty, environmental exposures, and healthcare quality and access, produce effects all of their own, independent of those exerted by each subsystem individually. With regard to breast cancer specifically, it is possible that the interactive effects between tobacco use, poor diet, and low physical activity result in a greater increase in breast cancer risk than the additive effects of each risk factor would indicate. These behavioral risk factors may also interact with the effects of ACEs to further increase breast cancer risk. Given the disproportionate impact of each of these environmentally conditioned risk factors on Black women, breast cancer disparities between Black and white women may be understood as an emergent effect of systemic racism, operationalized through racial residential segregation. Such emergent effects are generally robust to changes in a given subsystem.

### 3.2. Robustness

Established systems exhibit the property of robustness. This means that systems tend toward a state of equilibrium, and that that state of equilibrium will generally persist even in the face of significant disruptions within individual subsystems. The emergent effects of the system as a whole will ensure that the equilibrium is reasserted. In the case of racial disparities, this may explain the resistance of disparities to targeted interventions [4]. For example, a bias-reduction intervention targeting the healthcare subsystem that does not address risk factors related to chemical exposure, physical activity, or ACEs may not disrupt systemic equilibrium sufficiently to significantly reduce disparities. This is, in part, due to the fact that each of these subsystems is related to the others in complex ways.

### 3.3. Complexity

In systems theory, complexity is a formal designation which refers to a system in which there are a large number of component subsystems, and a large degree of interrelatedness between those component subsystems [54]. In the study of racial disparities in breast cancer, there are several layers of complexity: (1) the complexity of the problem itself; (2) the complexity of the interconnected web of causes that contribute to the problem; and (3) the complexity of the tasks associated with researching and intervening on the problem. For example, as mentioned above, behavioral risk factors, such as smoking, diet, and physical activity, may interact with one another in producing breast cancer risk. At the same time, they may also interact with social environmental exposures (i.e., violence, ACEs), or residential and occupational exposures to endocrine disrupting substances. Each of these layers of complexity contributes to breast cancer risk and to the intransigence of disparity. As mentioned above, complexity can pose a challenge for researchers and for interventionists, as parsimonious explanatory models and predictable results are difficult to achieve.

### 3.4. Leverage Points

In the context of complex systems, a full representation of all subsystems and all their interrelationships would yield a model insufficiently parsimonious for practical use. We propose that researchers, policymakers, and interventionists should conduct a critical review of the complex relationships between upstream causes of breast cancer and racial breast cancer disparities to identify systemic leverage points. Drawing on Reskin, we define leverage points as systemic nodes (or subsystems) where multiple other subsystems intersect, modifications in which have the potential to exert significantly amplified effects across the entire system [4]. As long as interventions to reduce racial breast cancer disparities confine themselves to only one or two subsystems within the broader context of racial inequity, the systemic quality of robustness will likely result in the reassertion of equilibrium, and the persistence of disparity. In contrast, intervention approaches to racial breast cancer disparities that explicitly address leverage points have the potential to impact numerous subsystems simultaneously and disrupt that equilibrium. Thus, leverage points should be understood as ideal areas of intervention and research.

#### Place as a Leverage Point in The System of Racial Discrimination

As Williams et al. state, “Residential segregation has been identified as a leverage point or fundamental causal mechanism by which institutional racism creates and sustains racial economic inequities” [5] (p. 117). Despite much intervention, residential racial segregation continues to be pervasive in the US. It should be noted, however, that racially homogenous communities are not innately problematic. Rather, the problem is that segregation is driven by racism. Racism has been defined as, “an organized societal system in which the dominant racial group uses its power to devalue, disempower, and differentially allocate societal resources and opportunities to groups defined as inferior” [55] (p. 2). For example, the Plessy v. Ferguson decision is correctly derided for upholding the constitutionality of segregation, and for maintaining the fiction that there could be “separate but equal” facilities for Black and white Americans. This decision was unjust precisely because of the reality that communities in which Black Americans lived, and their accompanying infrastructure, suffered from radical, intergenerational economic deprivation and social oppression. The consequences of that deprivation persist to this day, and they include disparities in health.

Given that residential segregation has facilitated the production of disparate health outcomes, “place” is of central importance in shaping health trajectories of individuals and communities. The relationship between place, race, and breast cancer is already the subject of attention from health scientists and interventionists. State level authorities and researchers are actively working to understand and address racism, social and environmental exposures, and inequities in community infrastructure, based on the understanding that each of these represent critical upstream factors that condition the distribution of an array of other risk factors for breast cancer [16,56]. In fact, racism has significant detrimental effects on economic development, physical infrastructure, and the social environment in communities of color. Therefore, programs that address residential segregation and identify place as a key leverage point will have the advantage of addressing each of these areas at once. Experiences of discrimination occur within the social and spatial context of the communities and workplaces within which people of color live, and these communities and workplaces disproportionately play host to the many risk factors related to social and physical environments outlined above. We argue that place is a systemic leverage point through which numerous other subsystems exert a deleterious effect on communities of color, and contribute to breast cancer disparities. Though this framing is by no means comprehensive, it does suggest that the effectiveness of efforts to reduce disparities could potentially be maximized through a focus on place, since it is a modifiable point at which all of the other systems intersect.

## 4. Directions for Future Research

There are three major areas of research that could help further the work of harnessing the power of place to reduce racial disparities in breast cancer. First, research is needed that traces the full causal pathway between the environment (both social and physical) and breast cancer outcomes. Second, further research is needed in the design and implementation of community-based (or place-based) intervention frameworks to address the upstream factors that contribute to racial breast cancer disparities in residentially segregated communities. Third, research should be conducted to explore the relationships between racial discrimination and other forms of discrimination as they pertain to breast cancer disparities.

Though a significant and growing body of literature traces the associations between numerous social, environmental, and biological factors and breast cancer incidence, the causal mechanisms that underlie the observed associations are often not well understood. For instance, though studies near uniformly show a positive association between breast density and breast cancer risk, the biological mechanism through which this association is operationalized is unknown [17]. It is possible that there is a confounding effect exerted by environmental factors associated with both breast density and breast cancer risk. Further, between and among the broad array of factors that are known to be associated with breast cancer risk, there are complex interrelationships that make rigorous assessment of a single causal mechanism extremely difficult. For example, communities of color tend to have lower SES than white communities. In those communities, risks of exposure to carcinogenic substances tends to be elevated, access to safe outdoor spaces conducive to physical activity is more limited, and quality foods are harder to come by [16,25,35,40,41,42].

There is a need to better understand how social factors, such as explicit and implicit discrimination, SES, occupational exposures, and access to nutrition, interact with factors in the built environment, such as chemical and other environmental exposures, light at night, and accessibility of green space, to exert influence on human physiology in ways that elevate breast cancer risk [16]. In turn, we must explore further how racial differences in the distribution of allostatic load, adverse epigenetic changes in the HPA axis, and endocrine disruption stemming from environmental stressors and exposures relate to racial disparities in breast cancer incidence and outcomes [5,49,50]. Given the elevated impact of certain environmental exposures on breast cancer risk during critical developmental phases early in life, intervening in the social and built environment is particularly promising for breast cancer prevention over the long term [16]. However, empirical investigation is needed to explore which environmental exposures drive breast cancer disparities, and what intervention approaches would be effective in reducing the risk they represent. Overall, more research is urgently needed on the ways in which social factors, such as poverty and violence, interact with correlated environmental exposures, such as industrial pollutants and residential chemical exposures, to influence breast cancer incidence in women of color. Tracing the full causal pathway from environment to incidence could inform the development of targeted multilevel programs to disrupt the equilibrium of the system of racial disparity.

According to Williams et al., “we lack the empirical evidence to identify which mechanisms of segregation (e.g., educational opportunity, labor market, housing quality) should be tackled first, would have the largest impact, and is most likely to trigger ripple effects to other pathways” [5] (p. 117). Several types of research endeavors could further this work, and systems thinking can be applied at numerous levels. For example, though we argue in this paper that, in the broader system of racial breast cancer disparities, “place” functions as a leverage point, it is possible to use the same process to identify other leverage points. Further, if “place” is selected as the key leverage point, and a specific community of practice is identified, there are likely further leverage points that should be identified within that community which function as highly interconnected nodes through which multiple subsystems exert influence on breast cancer disparities. These community-based leverage points may be community centers, health centers, the transportation system, the labor market, municipal authorities, or any number of other possibilities. Identifying these leverage points will require significant input from community stakeholders who can act as experts on the specific strengths and challenges of that community.

One possible area of research would therefore engage community stakeholders in the development of community-specific concept maps of key systemic interrelationships to identify leverage points, whereas another would pilot interventions informed by these maps to coordinate community efforts toward addressing those leverage points. When promising intervention frameworks are available, larger implementation studies should be carried out to determine the most effective strategies to ensure program adoption, fidelity, sustainment, and effectiveness [57]. Overall, a robust body of literature is needed that explores the complex interrelationships of subsystems contributing to breast cancer disparities, and tests the efficacy of place-based interventions to address key leverage points.

Though the focus of this discussion has been on racial discrimination as a determinant of breast cancer disparities, a third area of needed research would explore the intersectional relationships between racial discrimination and other forms of discrimination. For example, gender discrimination has been shown to be negatively associated with mammography screening, and it is plausible that this reduction in screening may disproportionately impact Black women [58]. Therefore, further research should be conducted exploring the intersectional impacts of racial and gender discrimination, and particularly the possible moderating impact of gender discrimination on the relationship between racism and breast cancer screening behaviors. This research may harness the power of place through community engagement approaches similar to those outlined under research areas one and two above.

## 5. Application to Breast Cancer Prevention

Eliminating racial breast cancer disparities will require significant community investment and coordinated efforts across multiple systems to prevent breast cancer in communities of color. The upstream causes of disparities are tied to place, and these efforts will need to be guided by community stakeholders to address community-specific concerns. In this paper, we provide a conceptual framework for one approach to harness the power of place to reduce breast cancer disparities. The potential applications of systems theory, and related leverage points, to breast cancer prevention are numerous. Traditional approaches tend to ignore these interrelationships in favor of linear models that are perceived to be more parsimonious and actionable. However, they often obfuscate the reality that emergent, systemic effects maintain the equilibrium of disparity, even in the face of significant investment in interventions targeting subsystems. Given that the various subsystems that make up the causal foundations of racial breast cancer disparities are spread across a diverse array of interrelated domains of practice and expertise, we argue that efforts to reduce these disparities should: (1) engage stakeholders across multiple sectors (subsystems), especially community members; (2) be informed by appropriate concept maps of the complex systems at play in the specific community; and (3) represent a coordinated effort across these sectors to address key leverage points at which multiple subsystems intersect.

If we are to offset the negative effects of systemic racism to reduce and ultimately eliminate racial inequities in health broadly, and breast cancer specifically, there must be a greater focus on creating “communities of opportunity” [55]. This term describes “the transformation of local communities (that had been historically disadvantaged because of racism and its related systematic under-investments), into places that provide opportunities in education, labor markets, housing markets, credit markets, health care and all other domains that drive well-being” [55] (p. 3). If investments are made to restructure place to provide equitable access to opportunities and resources, outcomes related to health and well-being will improve, disparities will be reduced, and a positive trajectory toward equitable outcomes can be established. An effective place-based initiative should focus on changing the systems to prioritize the provision of opportunities and resources that shape health and well-being to better serve the population. Such initiatives may include efforts aimed at early childhood development, improving neighborhood environments and housing conditions, reducing childhood poverty, enhancing income and employment opportunities, and access to high quality health care. Though any successful effort will require significant community investment, the costs should be offset by the fact that interventions on the upstream factors contributing to breast cancer disparities are likely to have a positive impact on health and welfare across multiple other domains, including cardiovascular and mental health, crime and violence, and overall quality of life. It is possible, through a process of strategic and sustained investment in historically marginalized communities, that intergenerational deprivation may be permanently eliminated, and the resultant disparities significantly reduced.

Finally, it should be noted that marginalized communities are not monolithic, and there is significant heterogeneity both between and among the various communities who experience health disparities. Taking a place-based, community-engaged approach to breast cancer prevention is therefore advantageous, given that it will allow for the development of research questions and intervention strategies that address the specific strengths and challenges of each community. Though much of the focus in this essay has been on racial breast cancer disparities between Black and white women, the methods outlined here have significant applicability in the task of eliminating disparities in Latinx, indigenous, and other racial/ethnic populations.
